# The Dental Law in Ohio

**Published:** 1881-12

**Authors:** 


### THE DENTAL LAW IN OHIO.

Two or three years ago the legislature of Ohio appointed a committee, to whom was assigned the duty of codifying the laws of the State. The manner in which this work was done has been very severely criticised, especially with reference to some laws. A signal instance of botchwork is presented in the work of the Commission in the codification--condensation--of the dental law. It has been condensed from six sections of four to twelve lines each to two little paragraphs, of four to five lines each. It is completely emasculated, the marrow is all taken out of it, and it is doubtful whether any court would attempt to try a case under it as it now stands. The matter certainly requires the attention of the profession of the State.

At the approaching meeting of the State Society, the subject should be fully considered, and such action taken as will lead to the restoration of the law, with perhaps some amendments. The law should be made as efficient as the law in any other State in the country. Ohio was the pioneer in the enactment of a mandatory law. Several States now have dental laws more strict in their requirements than the Ohio law ever was; and certainly the leader should keep abreast with any other.

It is very desirable that the State Society should do what it can to secure proper action by the legislature. Let it not be overlooked.

